# Incorporation of *Lactiplantibacillus plantarum* subsp. *plantarum* Dad‐13 Into Chocolate Processing: The Effect on Physical, Nutritional, and Probiotics Viability During Storage

**DOI:** 10.1155/sci5/5511985

**Published:** 2025-12-30

**Authors:** Titiek Farianti Djaafar, Tri Marwati, Anna Fajariyah, Nendyo Adhi Wibowo, Novia Nur Aini, Mifta Gatya, Imelda Damarwati, Hariya Amalina, Gabriela Belinda Aulia, Endang Sutriswati Rahayu, Tyas Utami, Rini Yanti, Ulyatu Fitrotin

**Affiliations:** ^1^ Research Center for Food Technology and Processing, National Research and Innovation Agency, Yogyakarta, Indonesia, brin.go.id; ^2^ Faculty of Agricultural Technology, Universitas Gadjah Mada, Yogyakarta, Indonesia, ugm.ac.id; ^3^ Center for Food and Nutrition Studies, Universitas Gadjah Mada, Yogyakarta, Indonesia, ugm.ac.id; ^4^ Center of Excellence for Probiotics, Universitas Gadjah Mada, Yogyakarta, Indonesia, ugm.ac.id

**Keywords:** cocoa, dadih, functional chocolate, local probiotics, microbiology

## Abstract

Chocolate with added functional value has become increasingly popular due to growing consumer health awareness. *Lactiplantibacillus plantarum* subsp. *plantarum* Dad‐13 isolated from *dadih* (fermented buffalo milk) is a potential probiotic strain exhibiting various health benefits. Probiotic chocolate was formulated by adding *L. plantarum* Dad‐13. The experiments were performed in triplicate. The safety parameters of the chocolate, including microbial and heavy metal contamination, were evaluated to ensure its safety for consumption. Changes in probiotic chocolate’s physical, nutritional, and microbial properties were compared to those of nonprobiotic chocolate. Storage trials were further conducted to better understand the viability of probiotics in the chocolate products. The chocolate used in this study was safe for consumption, as indicated by the low contamination levels. Chocolate supplemented with *L. plantarum* Dad‐13 had similar nutritional characteristics to nonprobiotic chocolate. However, the addition of probiotics slightly altered its physical characteristics, resulting in broader melting properties, although this remained within a tolerable range. Storing the chocolate at low to moderate temperatures (4–20°C) could maintain the viability of *L. plantarum* Dad‐13 above 8 log CFU/g for up to 30 days of storage, demonstrating its promising potential as a novel probiotic chocolate product.

## 1. Introduction

The market and industry have a high demand for increasing the functional value of food products, and the addition of probiotics has gained significant interest due to their beneficial effects on human health. Probiotic cells have been extensively used in various products, particularly dairy products and their derivatives. The incorporation of probiotics into food and beverages offers functional advantages, including enhancing gut health, boosting immune function, lowering cholesterol levels, and providing protection against cancer, particularly colon cancer [1, 2]. *Lactiplantibacillus plantarum* subsp. *plantarum* Dad‐13 (previously classified as *Lactobacillus plantarum* Dad‐13) is an indigenous probiotic isolated from dadih (traditional fermented buffalo milk from West Sumatra, Indonesia) (E S [3]). Previous studies have shown that *L. plantarum* can act as a probiotic in the gut, exhibiting antimicrobial properties against pathogens without translocating into the bloodstream or organs [4, 5]. *L. plantarum* Dad‐13, specifically, has been reported to exhibit various biological activities, including antimicrobial activity toward Enterobacteriaceae, *E. coli*, and other pathogens; regulate blood lipids and sugar levels; and improve nutrient absorption during digestion [4, 6]. Furthermore, *L. plantarum* Dad‐13 is suggested as likely to be safe for human consumption (E S [3]). Due to its beneficial properties, *L. plantarum* Dad‐13 has been used in various milk‐based products. However, its application on chocolate products is still limited.

Chocolate is a popular confectionery produced from cocoa beans. Chocolate products are popular due to their exceptional sensorial and functional properties. It contains a high amount of methylxanthines and polyphenols, which act as psychoactive compounds and antioxidants, and can reduce the risk of cardiovascular disease and cancer [1, 7]. Chocolate has unique textural properties, such as being solid at room temperature, snapping and melting in the mouth during consumption, and possessing high flavor release ability. These properties made chocolate an excellent carrier for functional ingredients, such as probiotics. However, incorporating probiotics in chocolate is challenging. Previous studies showed that chocolate is suitable as a probiotic carrier due to its high fat content, which can protect the probiotics from gastrointestinal digestion [8]. Additionally, it provides a long shelf‐life, ensuring more availability of viable probiotics [9]. However, it is highly dependent on the microorganisms used as probiotics [10]. The use of *L. plantarum* Dad‐13, specifically as a probiotic in chocolate, especially in terms of its physical and nutritional value, has never been studied. Understanding its behavior and viability in chocolate products is necessary to provide more information on its utilization in chocolate‐processing industries.

The formulation of chocolate products significantly affects their textural properties. The authors in [11] reported that adding probiotics to chocolate could alter its textural properties and lead to undesirable changes. Optimization in the probiotic chocolate processing is required to keep the changes tolerable. It is important to obtain probiotic chocolate, which not only boosts its functional value but can also provide a consuming experience to the customer. On the other hand, storage conditions also significantly affect the quality of probiotic chocolate. Previous reports on the development of probiotic chocolate showed varied responses of probiotics’ viability toward the temperature of storage [9, 12]. It was due to the differences in the tolerance and adaptability of the probiotic cells toward temperature. Hence, a study on the response and viability of *L. plantarum* Dad‐13 toward storage conditions is important to ensure its function as a probiotic in the chocolate product. To date, no study has been reported on the formulation of chocolate with *L. plantarum* Dad‐13 and the effect of storage on its viability. This study aimed to incorporate *L. plantarum* Dad‐13 into chocolate made from local cocoa beans. The formulation was evaluated for its textural, nutritional, and physical changes and its capability to maintain a viable number of probiotic cells during storage. The optimum storage condition that can maintain the textural, nutritional, and viability of probiotics is expected in this study.

## 2. Materials and Methods

### 2.1. Materials

Dried fermented cocoa beans (Forastero variety) for chocolate production were obtained from a local producer (Ngudi Raharjo II, Gunungkidul, Yogyakarta, Indonesia). Other ingredients were obtained from the local market, including sugar (PT. Madubaru–PG. Madukismo, Bantul, Yogyakarta, Indonesia), cocoa butter (Agricultural Technology Park, Gunungkidul, Yogyakarta, Indonesia), skim milk powder (PT. Mirota KSM, Sleman, Yogyakarta, Indonesia), whole milk powder, and milk butter (PT. Fonterra Brands Indonesia, Semarang, Indonesia). Probiotic powder of *L. plantarum* Dad‐13 with a concentration of 10^10^ CFU/g was obtained from the Food and Nutrition Culture Collection (FNCC, Universitas Gadjah Mada Yogyakarta, Indonesia). The probiotic powder was received in aluminum foil (KlinPak) packaging. The media and chemicals for analysis were of analytical grade.

### 2.2. Production of Probiotic Chocolate

A total of 5 kg cocoa beans were first steamed at 100°C for 20–30 min, followed by roasting at 120°C for 30 min to obtain roasted cocoa beans. The roasted beans were then deshelled and ground by using a winnower and a mechanical grinder to obtain chocolate liquor. The formulation was done by mixing chocolate liquor (25%), with cocoa butter (25%), sugar (25%), and full cream milk (20%), in a ball mill [13]. The refining and mixing process was carried out at 65°C for 6 h. The refined formulation was then conched at 50°C for 2 h. The final formula was then processed through the tempering process for nonprobiotic chocolate. For the probiotic chocolate, probiotic powder (5%) of *L. plantarum* Dad‐13 (10^10^ CFU/g) was added in the last 10 min of the conching process. Tempering was then done as follows: The formula was heated at 50°C for 9 min, then reduced to 27°C for 9 min and raised to 32°C for 9 min. The tempered formula was then put in the mold and subjected to the final tempering process in the chiller for 24 h. The molded chocolate was then packed in sealed aluminum foil packaging until analysis ([14] with a slight modification).

Samples were collected from every processing step, including cocoa beans, roasted cocoa nibs, chocolate liquor, and chocolate after tempering for the analysis of microbial and heavy metals contamination. Nutritional analysis was done on nonprobiotic (control) and probiotic chocolate after tempering. Samples for the physical characteristics and melting point analyses were prepared by storing the tempered chocolate in two different maturation conditions, namely, at room temperature (20°C) and in a chiller (4°C) for two weeks. On the other hand, the final probiotic chocolate product was also subjected to prolonged storage treatment (30 days) at various storage temperatures (4, 10, 20, 30, and 37°C) for the analysis of the cell viability.

### 2.3. Microbial Analyses

Microbiological contamination was evaluated according to SNI 7934:2014 [15]. The analyses included total plate count (TPC), *E. coli*, *Salmonella*, mold, and yeast contamination. The results were expressed as CFU/g for TPC, the most probable number (MPN)/g for *E. coli*, and colony/g for mold and yeast. The detailed analysis method was presented in Supporting Material Figure S1.

### 2.4. Heavy Metal Contamination Analysis

Heavy metal contamination was analyzed using the atomic absorption spectroscopy (AAS) method (Hasan and Faroque 2020). The determination of arsenic (As), cadmium (Cd), mercury (Hg), lead (Pb), and tin (Sn) was carried out by the wet ashing method using HNO_3_ and HClO_4_. Gradual heating was then carried out at temperatures of 130°C, 150°C, 170°C, and 200°C. The sample was then measured with absorbance measurements taken at its specific wavelength [16].

### 2.5. Analysis of the Nutritional Value of the Chocolate

Proximate analyses were done based on the AOAC method [17]. Moisture content was analyzed gravimetrically after drying in an oven (105°C, Medcenter Venticell, Schönwalde‐Glien, Germany) until a constant weight was obtained. Ash content was determined after ashing at 700°C for 3 h in a muffle furnace (Heraeus, Apeldoorn, Netherlands). Soxhlet extraction and the Kjeldahl method were used to determine fat and protein content, respectively. Sugar was analyzed using the dinitrosalicylic acid (DNS) method employing glucose as a calibration standard. Macrominerals were evaluated in digested samples by using a UV–Vis spectrophotometer (Shimadzu, Kyoto, Japan) at 766, 589, and 422 nm for potassium (K), sodium (Na), and calcium (Ca), respectively.

### 2.6. Physical Characteristics Analyses

The physical characteristics of chocolate were evaluated during the initial and final phases of the chocolate maturation period. Water activity (Aw) was determined using an Aw meter (Aqualab PAWKIT, Decagon Devices, Inc., Pullman, WA, USA) [18]. pH was measured using a pH meter (HANNA Hi98107, Leighton Buzzard, United Kingdom). The particle size of the chocolates was observed using a microscope and measured according to the method of Janovszky et al. [19] with modification. L∗, a∗, and b∗ color of chocolate was measured using a chromameter (Konica Minolta CR 400, Tokyo, Japan) according to Bahari and Akoh [20]. Hardness was evaluated using a universal testing machine (Zwick Z0.5, ZwickRoell Pte. Ltd, Boon Lay Wy, Singapore) [20]. The melting point was measured using differential scanning calorimetry (DSC, DSC‐60 Plus, Shimadzu, Kyoto, Japan) following the method of Janovszky et al. [19] with modification. Five milligrams of chocolate were hermetically sealed in a DSC pan and subjected to gradual heating from 0°C to 200°C (5°C/min) in the DSC apparatus. The results were presented in a melting curve, indicating the melting profile of the samples.

### 2.7. Probiotic Cell Viability Analysis

Cell viability was determined by the TPC method. Five grams of probiotic chocolate samples were dissolved with 45 mL of 0.85% NaCl in a stomacher. The solution was then subjected to dilution (three series) and plating in a pour plate of MRS agar. The plate was then incubated at 37°C for 24–48 h. The colonies formed were counted and expressed as log CFU/g [21].

### 2.8. Statistical Analysis

The experiments were performed in triplicate. Analysis of variance (ANOVA), Student’s *t*‐test, and Kruskal–Wallis analyses were done using SPSS 25 software (IBM Corp, Armonk, NY, USA).

## 3. Result and Discussion

### 3.1. Microbial and Heavy Metal Contamination

Microbial and heavy metal contamination are major concern in the safety of chocolate for consumption. Analysis of microbial contamination showed that the TPC, *E. coli*, *Salmonella*, mold, and yeast numbers were in the standard range for dried cocoa beans, roasted cocoa nibs, and chocolate liquor (Table 1). However, probiotic chocolate showed a relatively high TPC number (> 4.42 × 10^6^ CFU/g) both in liquor after tempering and in chocolate products. The addition of *L. plantarum* Dad‐13 culture during conching was the contributing factor elevating the TPC number in probiotic chocolate. PCA agar, as the media used for TPC analysis, was known to be a nonselective media for microbial growth [22]. Thus, the colony evaluated in the TPC analysis might have included the colony of probiotic cells. This was further confirmed by the result of probiotic cell viability. The result showed that the *L. plantarum* Dad‐13 cells were still active in probiotic chocolate after 30 days of storage. In this context, probiotic chocolate products should not be categorized and evaluated as conventional cocoa/chocolate products. The categorization of probiotic chocolate as a probiotic food product will be more appropriate to accommodate the rise of microbial counts due to probiotic enrichment.

**Table 1:** Microbiological contamination of cocoa products obtained from different stages of chocolate processing.

Microbiological contamination	Unit	SNI∗	Samples				
			S1	S2	S3	S4 (probiotic added	S5 (probiotic added)
Total plate count	CFU/g	max 1 × 10^4^	4.45 × 10^2^	6.73 × 10^3^	2.87 × 10^3^	5.83 × 10^6^	4.42 × 10^6^
*E. coli*	AMP/g	< 3 APM/g	Negative	Negative	Negative	Negative	Negative
*Salmonella*	—	Negative for each 25 g sample	Negative	Negative	Negative	Negative	Negative
Mold and yeast	Colony/g	max 1 × 10^2^	1.25 × 10^2^	< 1	< 1	0.7 × 10^2^	0.95 × 10^2^

*Notes:* Data were presented as means of triplicate. S1 = dried cocoa beans; S2 = cocoa nib; S3 = chocolate liquor; S4 = probiotic chocolate liquor after tempering; S5 = probiotic chocolate. ∗Based on SNI 7934:2014 about chocolate and chocolate products [15].

Abbreviation: SNI = *Standar Nasional Indonesia* (National Indonesian standard).

There was an increase in the microbial count during chocolate processing as evaluated by TPC analysis. Cocoa beans used in this study had a TPC number of 4.45 × 10^2^ CFU/g and increased to 6.73 × 10^3^ CFU/g after roasting and deshelling/winnowing. Roasting of cocoa beans could kill most of the microorganisms due to intense heat treatment [23]. However, the deshelling/winnowing process allowed the contact of cocoa nibs with air during the separation of shells and nibs. This increased the possibility of cross‐contamination of microbes from the air and increased the TPC number of cocoa nibs. On the other hand, chocolate liquor had a lower TPC number (2.87 × 10^3^ CFU/g) than that of cocoa nibs. Heating during the grinding, refining, and conching processes could help eliminate the microorganisms in cocoa products. Similarly, the heating treatment ensured the absence of *E. coli* and *Salmonella* (Table 1). *E. coli* is a temperature‐sensitive microorganism with optimal growth temperature between 35°C and 40°C. The increase in temperature to 50°C could eliminate it [24]. Furthermore, chocolate processing is a water‐free process. This ensured limited *E. coli* contamination of the chocolate product [25]. On the other hand, *Salmonella* was not found in any cocoa product in this study. The transmission of *Salmonella* to humans from chocolate products could be caused by the use of contaminated animal and plant‐based ingredients such as milk and nuts [26]. *Salmonella* is a dangerous food‐borne pathogen causing morbidity, mortality, and burden disease upon consumption [27]. Considering the danger, *Salmonella* should not be present in all food products, including chocolate and its derivative products [15].

Mold and yeast counts were relatively close to the standard limit (0.7 and 0.95 × 10^2^ colony/g). Mold contamination is commonly found in cocoa beans, especially in poor postharvest treatment. In this study, the highest number of molds was found in dried cocoa beans. This might be caused by the long drying period of cocoa beans after fermentation. Cocoa beans used in this study were sundried and may have experienced interrupted drying due to humid conditions. This condition allowed the inside of cocoa beans to be susceptible to mold growth. The mold found in the samples belonged to the *Aspergillus* and *Fusarium* groups. Their number was slightly reduced after the grinding, refining, and conching. Molds can survive in extreme conditions such as low pH and Aw content [28]. This is contributed by the ability of molds to form heat‐resistant spores. Even though the number of molds and yeast in probiotic chocolate was below the allowed standard, postharvest practices of cocoa beans need to be thoroughly evaluated. The occurrence of molds and yeast in chocolate may result in altered sensory properties due to their ability to ferment the sugar in the chocolate.

**Figure 1:**
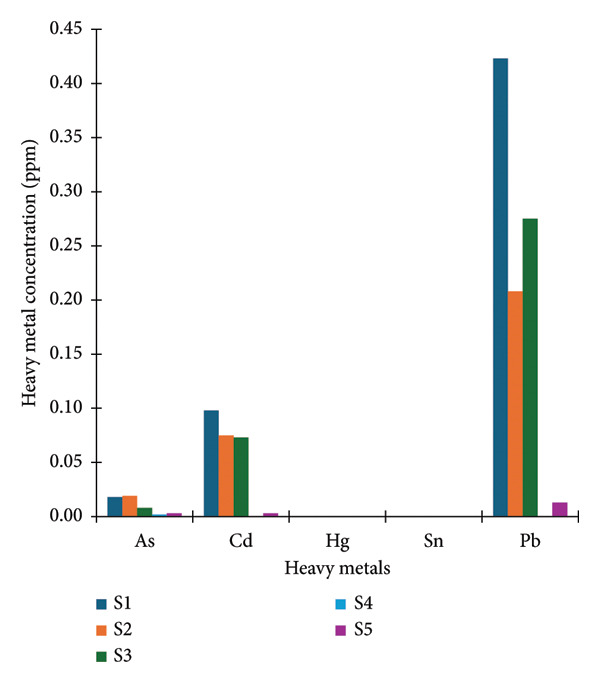
Heavy metals’ concentration in dried cocoa beans, cocoa nib, chocolate liquor, and probiotic chocolate.

The concentration of heavy metal contamination in cocoa products in all stages of the process remained within the limits specified in the standard [15] (Figure 1 and Table 2). Arsenic, cadmium, and lead are heavy metals commonly found in the material components of volcanic eruptions. They can contaminate soil, rocks, water, and air [29]. The cocoa beans used in this research were obtained from Bunder village, Patuk subdistrict, Gunungkidul, Yogyakarta, Indonesia. The soil in this area is mostly latosol soil containing volcanic rocks [30]. Thus, the contamination might have occurred during the cocoa plantation due to its soil condition. The concentration of As, Cd, and Pb was reduced during chocolate processing. This was in agreement with the result of Bayraklı et al. [31]. The addition of other ingredients reduced heavy metal concentrations due to differences in the total amount of ingredients in the final product. Meanwhile, cocoa beans and the final product of probiotic chocolate were free from Hg and Sn contamination. Based on these results, it was concluded that the probiotic chocolate in this study was safe for consumption due to low contamination of microbial and heavy metals.

**Table 2:** The Indonesia National Standard concentration of heavy metal.

Heavy metal	Unit	SNI^∗^
As	ppm	1
Cd	ppm	0.5
Hg	ppm	0.03
Sn	ppm	40
Pb	ppm	1

Data are presented as means ± standard deviation. S1: dried cocoa beans; S2: cocoa nib; S3: chocolate liquor; S4: probiotic chocolate liquor after tempering; S5: probiotic chocolate; ^∗^based on SNI 7934:2014 about chocolate and chocolate products [15]. Abbreviations: ND, not detected; SNI, *Standar Nasional Indonesia* (Indonesian standard); As, arsenic; Cd, cadmium; Hg, mercury; Sn, tin; Pb, lead.

The addition of probiotics, particularly lactic acid bacteria, has been shown to reduce heavy metal content in chocolate products. This mechanism is presumed to be associated with the ability of probiotic cells to adsorb metal ions, as well as the production of metabolites such as lactic acid and exopolysaccharides that precipitate metals into insoluble salts [32]. Consequently, heavy metals are less detectable in their free form and their bioavailability decreases. Upon consumption, metals bound to probiotics are poorly absorbed in the intestine and are eliminated through feces, thereby contributing to a reduction in toxicity risk. Supported by previous papers, the various strategies adopted to reduce the harmful effects of heavy metals by L. plantarum in vivo to reduce heavy metals include promoting the excretion of heavy metals and reducing their content in the tissues. *L. plantarum* exhibited a significant effect in reducing the blood concentration of methylmercury, and results of in vivo experiments in mice have shown that *L. plantarum* CCFM8610 could effectively reduce the cadmium content in mice exposed to cadmium and promote the excretion of cadmium through feces [33, 34].

### 3.2. Nutritional Value of Chocolate Products

The nutritional value of probiotic chocolate was similar to that of nonprobiotic (Table 3). This was reasonable since they shared similar ingredients and formulations. Compared to the nutritional values of commonly consumed chocolate products based on USDA [35], chocolate products in this study had lower moisture, sodium, and calcium content and higher protein, fat, and potassium content. These differences might be attributed to the different formulations of chocolate products. Chocolate is a highly customized product. It can be formulated from various ingredients to meet consumer needs. Thus, it was reasonable that the nutritional values of chocolate products in this study differed greatly from those of the market.

**Table 3:** Nutritional values of probiotic chocolate and nonprobiotic chocolate compared to USDA data.

Parameter	Nonprobiotics	Probiotics	USDA 2016^∗^
Moisture content (%)	2.81 ± 0.275^a^	2.82 ± 0.357^a^	5.3097
Ash content (%)	1.52 ± 0.149^a^	1.48 ± 0.093^a^	—
Protein content (%)	8.33 ± 0.535^a^	8.34 ± 0.319^a^	2.6549
Fat content (%)	46.98 ± 1.106^a^	47.51 ± 1.429^a^	12.3893
Sugar content (%)	23.41 ± 0.767^a^	23.87 ± 0.976^a^	23.0088
Sodium content (mg)	101.46 ± 23.435^A^	106.68 ± 42.623^A^	129.2035
Potassium content (mg)	426.02 ± 21.967^A^	492.15 ± 90.245^A^	179.646
Calcium content (mg)	128.01 ± 16.886^A^	123.46 ± 1.550^A^	90.2654

*Note:* Data are presented as means ± standard deviation. Values with the same lowercase letter were not significantly different based on the independent sample *t*‐test (*p* > 0.05); values with the same uppercase letter were not significantly different based on the Kruskal–Wallis test (*p* < 0.05).

Abbreviations: T = temperature; USDA = United States Department of Agriculture.

^∗^U.S. Department of Agriculture [35].

The addition of probiotics did not alter the nutritional values of chocolate in this study (*p* > 0.05). This showed the versatility of probiotics *L. plantarum* Dad‐13 as a functional ingredient for food products. The incorporation of probiotics in food products usually leads to changes in the nutritional component, as reported by the authors in [36]. This is due to the metabolism of probiotic bacteria through enzymatic activity to degrade protein, carbohydrate, and fat. No alteration in the nutritional component might also refer to no alteration in sensory properties. This is important since chocolate is a product that serves as a pleasurable function. Hence, the alteration of chemical and sensorial properties in chocolate after production is undesirable. Furthermore, the incorporation of probiotics in chocolate might offer another advantage. A previous study by the authors in [21] showed that high‐fat content in food such as chocolate could protect probiotics during gastrointestinal digestion, ensuring their higher bioaccessibility after consumption. This condition made chocolate an ideal carrier for probiotics, especially *L. plantarum* Dad‐13.

### 3.3. Physical Characteristics of Probiotic Chocolate

As a product for hedonic consumption, the physical characteristics of chocolate are an important quality parameter. These characteristics affect the shelf‐life, appearance, and textural properties of chocolate. In the last stage of chocolate production (Week 0), there were no differences observed between probiotics and nonprobiotic chocolate (Table 4). They shared similar physical properties. However, their physical characteristics were altered after the maturation period (Week 2), showing significant differences in Aw, pH, whiteness index, and hardness. In this study, the changes observed were dependent on the maturation temperature. The maturation process at 20°C led to greater changes in the evaluated parameters. Aw is a crucial parameter determining product stability during storage (Tapía, *et al.*, 2020). It is related to the appearance and shelf‐life of food ingredients. The overall Aw value ranges from 0.47 to 0.58. This is consistent with the research by Djaafar et al. [37], who reported that the Aw in chocolate was 0.68. Therefore, the Aw value is still within the appropriate range to prevent the growth of molds. On the other hand, a decrease in the pH of chocolate after maturation was observed. This may be due to fat degradation. The result of this study was in agreement with the result of Dalheim et al. [38], who reported that the pH value of chocolate decreased due to the presence of H ions due to oxidation. The rate of fat oxidation reaction at a temperature of 20°C was faster than that at 4°C [38]. This was in agreement with the result of this study.

**Table 4:** Comparison of the physical characteristics of probiotic and nonprobiotic chocolate before and after the maturation period.

Physical characteristics	Temperature of maturation (°C)	Week 0		Week 2	
		Probiotics	Nonprobiotics	Probiotics	Nonprobiotics
Water activity	4	0.47 ± 0.00^A^	0.47 ± 0.01^A^	0.50 ± 0.01^aB^	0.51 ± 0.01^aB^
	20	0.47 ± 0.00^A^	0.47 ± 0.01^A^	0.56 ± 0.01^aC^	0.58 ± 0.02^aC^
pH	4	5.66 ± 0.01^A^	5.61 ± 0.06^A^	5.27 ± 0.04^aB^	5.29 ± 0.07^aB^
	20	5.66 ± 0.01^A^	5.61 ± 0.06^A^	5.10 ± 0.02^aC^	5.12 ± 0.08^aC^
Particle size (μm)	4	10.57 ± 0.25^A^	10.56 ± 0.54^A^	11.00 ± 0.24^aA^	11.09 ± 0.43^aA^
	20	10.57 ± 0.25^A^	10.56 ± 0.54^A^	10.25 ± 0.10^aA^	10.21 ± 0.22^aA^
Whiteness index (%)	4	32.82 ± 0.05^A^	32.46 ± 0.09^A^	33.93 ± 0.84^aB^	33.53 ± 1.12^aB^
	20	32.82 ± 0.05^A^	32.46 ± 0.09^A^	33.82 ± 1.00^aB^	34.02 ± 0.10^aB^
Hardness (N)	4	42.85 ± 1.19^A^	43.77 ± 2.40^A^	45.86 ± 0.01^aA^	45.76 ± 0.01^aA^
	20	42.85 ± 1.19^A^	43.77 ± 2.40^A^	33.82 ± 0.01^aB^	33.68 ± 0.02^aB^

*Note:* Data were presented as means ± standard deviation. Values with different lowercase letters in the same row were significantly different (*p* < 0.05). Values with different uppercase letters in the same category of physical characteristics were significantly different (*p* < 0.05).

No significant changes in particle size were observed in chocolates before and after the maturation. Particle size is determined by the processing method and the duration of the refining process. The particle size of chocolates ranged from 0.6 μm to 300 μm at the beginning and reduced to 0.6–150 μm at the end of the process [39]. In this study, probiotic and nonprobiotic chocolates were refined using the ball mill for 12 h at 65°C, resulting in particle sizes less than 20 μm. This ensured the smooth texture of probiotics and nonprobiotic chocolate produced in this study.

Whiteness index and hardness were the most noticeable changes observed in the chocolate in Week 0 and after maturation (Week 2). Maturation in chiller temperature (4°C) improved appearance and textural properties. Both chocolates (probiotics and nonprobiotics) had a brighter appearance after maturation. This was in agreement with Janine et al. [40] and Toker et al. [41], who found that a smaller particle size in chocolate due to the tempering and maturation process leads to a larger specific surface area, creating more particle interactions. This caused the chocolate particles to be denser and emit more light, resulting in a brighter color [40]. Comparison of visual appearance between probiotic and nonprobiotic chocolate in Week 0 and Week two of storage is shown in Figure 2. In Week 0, probiotic and nonprobiotic chocolate have the same visual appearance. Moreover, the appearance of white crystals indicated that blooming had occurred after maturation (Week 2). The probiotic and nonprobiotic showed the same presence of white crystals.

Figure 2Comparison of the visual appearance between probiotic and nonprobiotic chocolate at Week 0 and Week 2 of storage. (a) Probiotic chocolate Week 0. (b) Probiotic chocolate Week 2. (c) Nonprobiotic chocolate Week 0. (d) Nonprobiotic chocolate Week 2.

**Figure:**
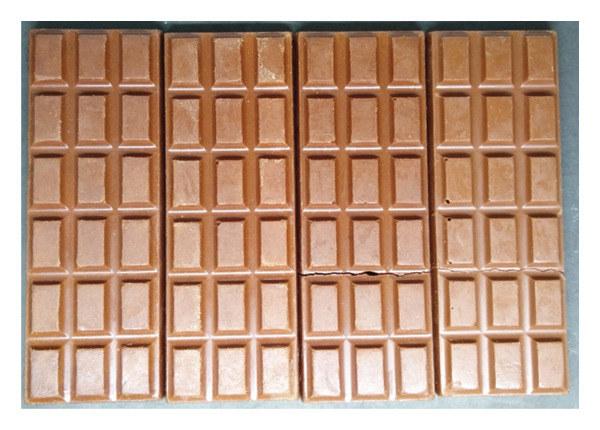
(a)

**Figure:**
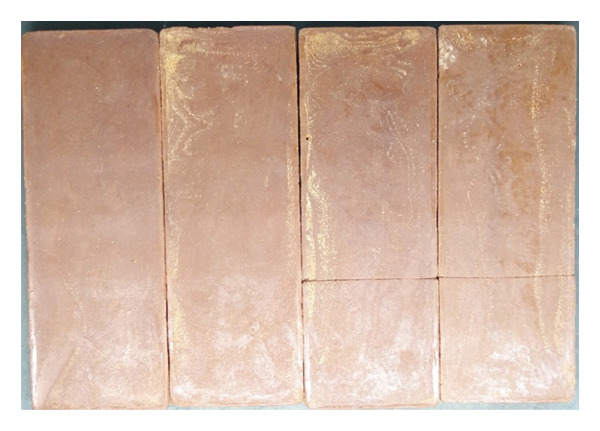
(b)

**Figure:**
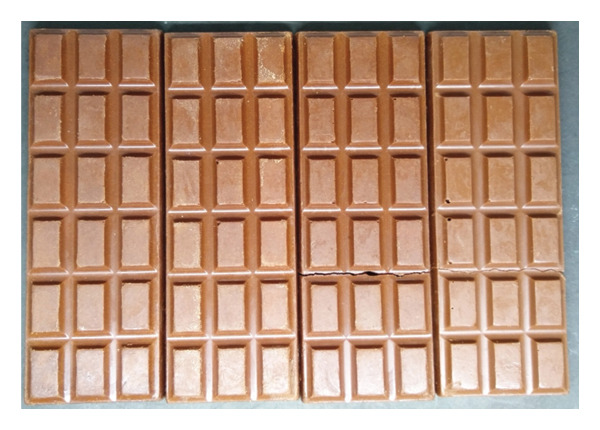
(c)

**Figure:**
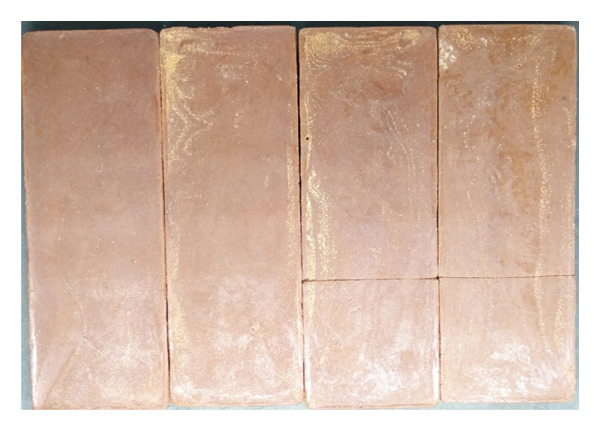
(d)

The hardness of all chocolate products was slightly increased after maturation at 4°C but greatly decreased when stored at 20°C. Hardness is an important parameter that provides snap properties, especially in couverture chocolate. Saputro et al. [42] reported that the water content and particle size of chocolate influence hardness. On the other hand, Deou et al. [40] reported that chocolate’s hardness value was affected by both water and fat content. Higher water content led to higher hardness values due to the formation of sugar networks [40]. The stickiness occurs on the surface of sugar particles in the chocolate, causing them to bond tightly and form a strong sugar network, increasing the hardness [42]. Considering that there was no contact with water during the maturation process, the decrease in hardness is more likely to be caused by the deformation of crystal properties in the chocolate. Storage at 20°C melted unstable cocoa butter crystals in chocolate, reducing the hardness due to looser particle interactions. The changes in fat crystal formation were confirmed by the result of melting point analysis using DSC.

Ideally, chocolate is solid at room temperature (20–25°C) and melts quickly at 33–34°C, close to the body temperature of around 37°C [43]. The taste and texture of chocolate are determined by the melting point properties of the fat crystals and the chocolate’s rheological and textural properties [40]. The ability of chocolate to quickly melt in the mouth is indicated by the sharp melting peak and short onset to endset temperature. In chocolate, the formation of beta (β) fat crystals (Types V and VI) is desired due to their ability to melt quickly at body temperature (33.8–36.3°C) [44]. In this study, nonprobiotic chocolate stored at 4°C had a short and sharp melting point (30.55°C–33.58°C), albeit lower than that of the previous study [44] (Figure 3 and Table 5). However, the short‐range melting point evaluated in that chocolate indicated that the tempering process was a success in producing uniform fat crystals, providing quick‐melt properties. On the other hand, nonprobiotic chocolate stored at 20°C showed slightly different melting properties than that stored at 4°C. This chocolate had a broader range of melting points (29.89°C–35.93°C), indicating the occurrence of various fat crystals, presumably Types IV, V, and VI. The occurrence of Type VI crystal provided a sharp melting point when exposed to high temperatures (Figure 2(d)). While the chocolate could still maintain its hardness, the melting profile during consumption might be different from that of well‐tempered chocolate.

Figure 3Differential scanning calorimetry (DSC) melting curves of chocolate products after ripening. (a) Nonprobiotic, 20 ^°^C. (b) Nonprobiotic, 4 ^°^C. (c) Probiotic, 20 ^°^C. (d) Probiotic, 4 ^°^C.

**Figure:**
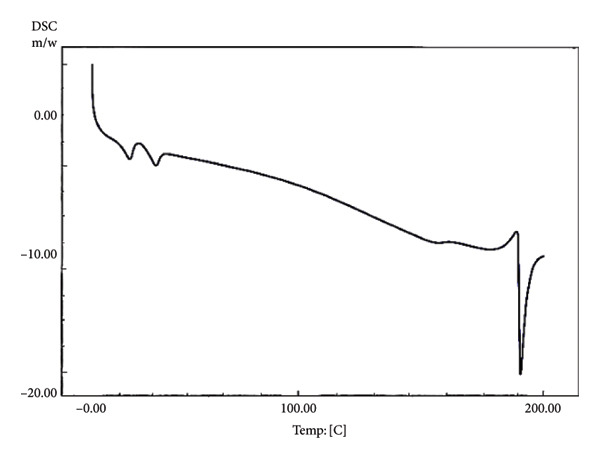
(a)

**Figure:**
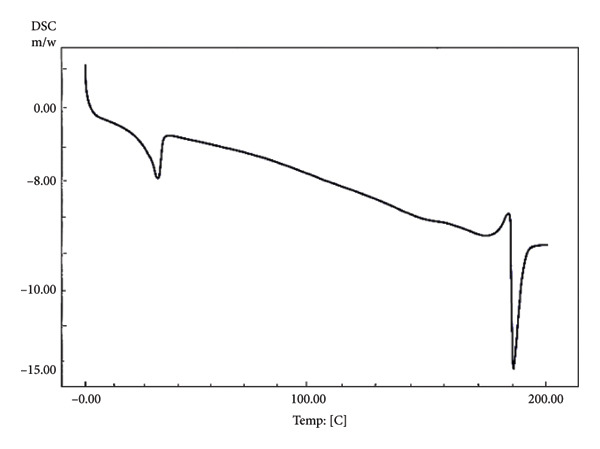
(b)

**Figure:**
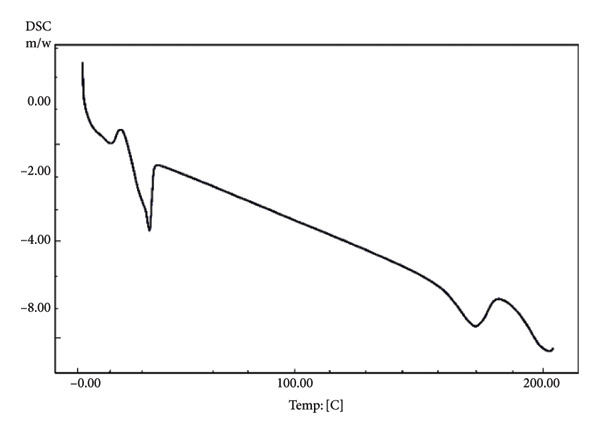
(c)

**Figure:**
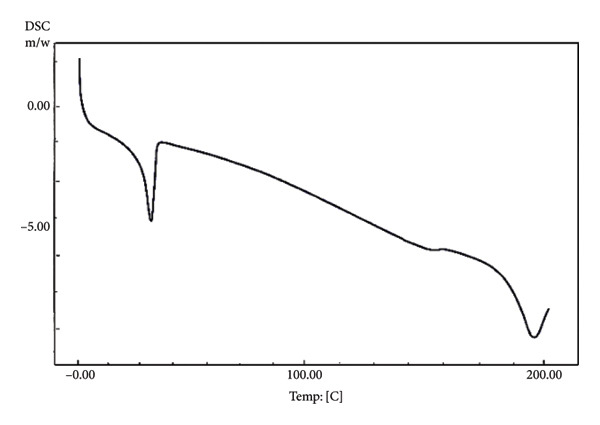
(d)

**Table 5:** Melting points during storage of probiotic chocolate compared to nonprobiotic chocolate.

Physical characteristics	Temperature of storage (°C)	Chocolate samples					
		Probiotics			Nonprobiotics		
		Onset (°C)	Peak (°C)	Endset (°C)	Onset (°C)	Peak (°C)	Endset (°C)
Melting point	4	24.39	30.77	32.93	30.55	31.90	33.58
	20	27.72	32.68	35.55	29.89	33.41	35.93

*Note:* Values were based on the DSC data.

The addition of probiotics slightly altered the melting properties of chocolate. Probiotic chocolates had a broader melting point compared to nonprobiotic. Probiotic chocolate stored at 4°C started to melt at a temperature range of 24.39–32.93°C. DSC melting curves of this chocolate showed eutectic behavior (Figure 2). This might correspond to the occurrence of two different phases of fat crystals, such as low–melting‐point alpha (*α*) crystals (Type II), moderate–melting‐point beta prime (Type IV), and beta (Type V). The coexistence of two different fat phases indicated that the addition of probiotics interfered with the tempering process. On the other hand, probiotic chocolate stored at 20°C had altered melting properties similar to those stored at 4°C. It had slightly broader melting points (27.72°C–35.55°C) compared to that of a nonprobiotic one stored at the same temperature (29.89°C–35.93°C). Eutectic behavior was also evaluated in this sample. Different from that of probiotics stored at 4°C, the eutectic curve occurred close to the melting point’s peak, indicating the occurrence of fat crystals with similar melting temperatures. However, the melting point peak was still in the range of 30–33°C. This was in agreement with the result of the authors in [45], who reported that the chocolate was solid at temperatures below 20°C, and it began to melt between 30 and 32°C and was fully melted at 35°C.

The addition of the probiotics and low‐temperature storage treatment was found to interfere with the tempering process of chocolate. On the other hand, the differences in the melting properties compared to other studies may also be attributed to the differences in the composition of fatty acids and triacylglycerols in cocoa butter. Thus, further optimization of the tempering process of probiotic chocolate is needed to ensure that the desired textural characteristics of chocolate can be achieved. The addition of probiotics during chocolate processing was carried out at the tempering stage at 30°C–40°C, ensuring that cell viability was not affected.

The changes in hardness and melting properties of probiotic chocolate can be addressed through process and formulation optimization. Previous studies have reported several strategies to minimize disruptions in cocoa butter crystallization caused by the presence of live cultures, including (1) probiotic microencapsulation, which can reduce direct interactions with the fat phase and stabilize fat polymorphism while maintaining the desired textural attributes [46]; (2) adjusting the levels of emulsifiers such as lecithin or PGPR, which has been shown to decrease hardness by up to 10%–20% through improved fat crystal dispersion [47]; (3) optimizing tempering parameters, particularly cooling rate and seeding, which play a crucial role in stabilizing βV crystals responsible for the characteristic snap and rapid melt‐in‐mouth properties of well‐tempered chocolate [44]; and (4) adding cocoa butter in appropriate amounts, which can narrow the melting range without compromising probiotic viability [48]. In this study, *L. plantarum* Dad 13 was used in encapsulated form; however, to shorten the melting point range, steps as mentioned above were required. Therefore, both formulation adjustments and process optimization offer promising approaches to overcome textural changes induced by probiotic incorporation and low‐temperature storage, ensuring that probiotic chocolate can still meet the expected sensory standards of conventional chocolate.

Based on previous reports, the sensory quality and consumer acceptance of probiotic chocolate containing *L. plantarum* Dad‐13 have been comprehensively evaluated [14]. It demonstrated through a controlled sensory evaluation using a five‐point hedonic scale that the addition of *L. plantarum* Dad‐13 in freeze‐dried powder form did not significantly affect color, aroma, taste, texture, or overall acceptance compared with nonprobiotic chocolate (*p* > 0.05). To complement these findings, a market acceptance study was conducted, involving more than 200 participants across Indonesian regions (such as Lombok Timur, Bali, Surabaya, and Yogyakarta), representing diverse consumer groups including children, university students, office employees, and housewives. Participants evaluated the probiotic chocolate using a structured acceptance questionnaire, and more than 80% rated the product positively (“*like*” to “*very like*”) and expressed willingness to purchase if the products were commercially available. These findings collectively indicate that the incorporation of *L. plantarum* Dad‐13 does not negatively affect the sensory properties of the chocolate and that the product demonstrates a high level of acceptance across different demographic groups and geographical regions, supporting its market potential as a functional food product.

### 3.4. Effect of Storage Temperature on Cell Viability of Probiotic *L. plantarum* Dad‐13 in Chocolate

The probiotic ingredient incorporated in this study was a locally isolated strain, *L. plantarum* Dad‐13, which was prepared in powdered form through freeze‐drying before use. This preparation method ensures stability and viability during storage while maintaining functional properties’ characteristics of the strain. Through several studies, *L. plantarum* Dad‐13 has been comprehensively validated through genomic, in vitro, and in vivo studies for its capabilities as a probiotic. Genome analysis revealed that this strain carries genes encoding acid tolerance (dltA, gadA, and gadB), bile salt hydrolase, and stress‐response proteins that facilitate survival during passage through the digestive tract (E S [49]). In vitro assays confirmed these genetic traits, showing that *L. plantarum* Dad‐13 maintains high viability in simulated gastric juice (pH 2‐3 with pepsin) and bile salt concentrations up to 3%, with minimal log reduction after 2–4 h exposure, indicating acid and bile tolerance consistent with probiotic requirements [50]. Importantly, when processed into a freeze‐dried powder, Dad‐13 retained high viable counts and showed excellent stability during storage, with its antibacterial activity preserved and survival maintained under simulated digestion conditions [51]. In vivo studies in Sprague–Dawley rats given high oral doses (10^11^ CFU/day) showed increased fecal recovery of *L. plantarum* and absence of translocation to blood or internal organs, confirming safety and gastrointestinal transit survival (E S [3]). Furthermore, a randomized, double‐blind clinical trial in undernourished children in Lombok demonstrated that daily consumption of chocolate containing *L. plantarum* Dad‐13 for 90 days significantly improved gut microbiota composition, increasing the relative abundance of beneficial taxa, such as Bacteroidetes and Bifidobacterium, and reducing fecal pH, indicating the health functional activity of the probiotic (E [49]). Collectively, these findings provide strong evidence that *L. plantarum* Dad‐13 survives the gastrointestinal environment and reaches the intestine in a viable, metabolically active form, fulfilling FAO/WHO criteria for probiotics.

**Figure 4:**
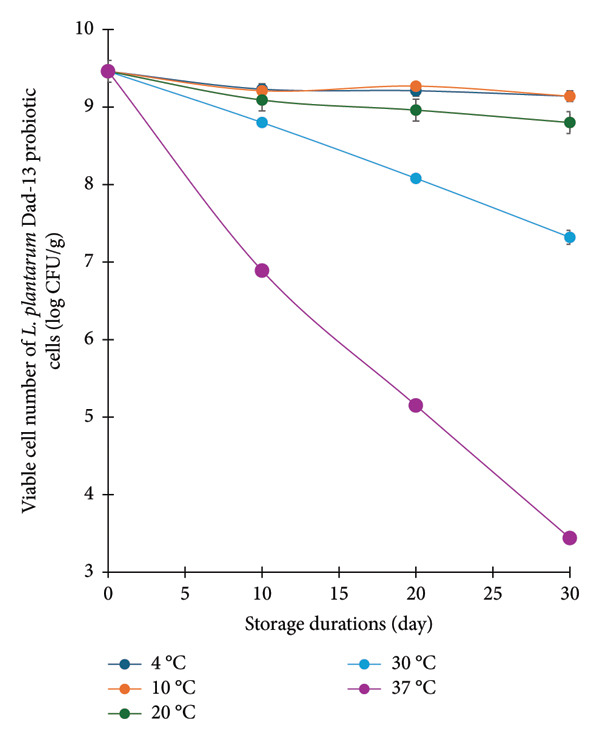
Viable cell number of *Lactiplantibacillus plantarum* Dad‐13 probiotic cells (log CFU/g) in probiotic chocolate at different storage durations and temperatures.

The viability of probiotic cells is essential in probiotic‐based products. To positively contribute to gastrointestinal health, the viability of probiotic cells in probiotic products must be at least 6 log CFU/g [2]. In this study, the viability of *L. plantarum* Dad‐13 decreased during storage, dependent on the temperature (Figure 4). Low to moderate temperature storage (4, 10, and 20°C) could maintain the viability of *L. plantarum* Dad‐13 (8‐9 log CFU/g) until 30 days of storage. This was in agreement with Hossain et al. [21], who reported that the cell viability of *Lactobacillus delbrueckii* subsp. *bulgaricus* remained above 7.5 log CFU/g after being stored in chocolate at 4°C and 25°C for 120 days. Similarly, microencapsulated *S. thermophilus* showed high viability (> 9 log CFU/g) in milk and dark chocolate stored for 180 days at 4°C [52]. The use of low temperature storage is beneficial to maintain the probiotics.

An increase in storage temperature results in a decrease in probiotic cell viability. High‐temperature storage (30°C and 37°C) significantly reduced the number of viable cells. High temperature accelerated the oxidative stress caused by the interaction of oxygen with proteins, nucleic acids, and lipids. Furthermore, the structural breakdown during high‐temperature storage, such as the melting of chocolate, may eliminate the protective function of the fat matrix, making probiotic cells susceptible to oxidative stress. This condition damaged the cell membrane and led to the death of probiotic cells [53].

### 3.5. Implications of the Results of the Study for Industrial and Commercialization Practices

This study successfully incorporated the probiotic *L. plantarum* Dad‐13 into the chocolate product. Even though some differences were observed in the physical characteristics of probiotic chocolate compared to those of nonprobiotic chocolate, their nutritional values were similar. Furthermore, the addition of *L. plantarum* Dad‐13 might increase the functionality of chocolate as a functional food product. From this study, we found that the addition of probiotics in chocolate resulted in interference in the microbial analysis related to food safety requirements, such as TPC. Cocoa beans are the product of fermentation. Hence, the occurrence of microorganisms is expected. Control and evaluation of these microorganisms are important to ensure the safety of chocolate products. For the production of probiotic chocolate, it is suggested that the sterilization of cocoa beans is carried out sufficiently to eliminate pathogenic microorganisms.

The addition of probiotics led to significant textural changes in the chocolate. In this study, the chocolate products evaluated were in the form of chocolate bars. As popular chocolate products boosted their textural quality, chocolate bars with improper snap and melting properties were regarded as low quality. Further study in the optimization of the tempering process of probiotic chocolate is needed to obtain probiotic chocolate with good textural properties. On the other hand, storage at low temperatures also led to changes in the melting profile of probiotic chocolate. This was problematic since probiotic chocolate needs to be stored at low temperatures to maintain its probiotic viability. A solution to maintain the textural properties of tempered chocolate without being affected by the addition of probiotics and storage in low temperatures is urgently needed. This may be done by adding stabilizers, such as vegetable fat or nonfat stabilizers. Furthermore, this opens the possibility of forming probiotic chocolate in no‐bar forms, such as truffle, praline, spread, or another form of product where the chocolate does not need to be tempered and can be stored at low temperatures.

## 4. Conclusion

The addition of *L. plantarum* Dad‐13 as a probiotic in chocolate could produce chocolate with good physical, nutritional, and functional properties. The textural properties of probiotic chocolate differed slightly from those of nonprobiotic chocolate. Chocolate was also proven to be a suitable carrier for probiotics due to its ability to protect and maintain its viability during storage. Further study needs to be done on the tempering process and the development of alternative forms of probiotic chocolate. Considering its functionality, probiotic chocolate based on *L. plantarum* Dad‐13 can potentially be developed as functional chocolate.

## Practical Application

The incorporation of *L. plantarum* subsp. *plantarum* Dad‐13 into chocolate products is an innovation and a probiotic delivering agent. The innovation of probiotic chocolate can provide insight into the industry to develop attractive snacks for consumers.

## Disclosure

(a) Patent: *Lactobacillus plantarum* Dad‐13 probiotic chocolate and its manufacturing process, 2022 (IDP000082524) (in Indonesian). (b) Patent: *Lactobacillus plantarum* Dad‐13 probiotic chocolate candy formulation, 2023 (IDP000089640) (in Indonesian).

## Conflicts of Interest

The authors declare no conflicts of interest.

## Author Contributions

**Titiek Farianti Djaafar:** conceptualization, data curation, formal analysis, funding acquisition, investigation, methodology, project administration, resources, software, supervision, validation, visualization, writing–original draft preparation, and writing–review and editing. **Tri Marwati:** conceptualization, data curation, formal analysis, funding acquisition, investigation, methodology, project administration, resources, software, supervision, validation, visualization, writing–original draft preparation, and writing–review and editing. **Anna Fajariyah:** data curation, formal analysis, funding acquisition, investigation, methodology, project administration, resources, software, validation, visualization, writing–original draft preparation, and writing–review and editing. **Nendyo Adhi Wibowo**: data curation, formal analysis, funding acquisition, investigation, methodology, project administration, resources, software, validation, visualization, writing–original draft preparation, and writing–review and editing. **Novia Nur Aini:** data curation, formal analysis, investigation, methodology, resources, software, validation, visualization, writing–original draft preparation, and writing–review and editing. **Mifta Gatya:** data curation, formal analysis, investigation, methodology, resources, software, validation, visualization, writing–original draft preparation, and writing–review and editing. **Imelda Damarwati:** data curation, formal analysis, investigation, methodology, resources, software, validation, visualization, writing–original draft preparation, and writing–review and editing. **Hariya Amalina:** data curation, formal analysis, investigation, methodology, resources, software, validation, visualization, writing–original draft preparation, and writing–review and editing. **Gabriela Belinda Aulia**: data curation, formal analysis, investigation, methodology, resources, software, validation, visualization, writing–original draft preparation, and writing–review and editing. **Endang Sutriswati Rahayu:** conceptualization, funding acquisition, supervision, and writing–review and editing. **Tyas Utami:** conceptualization, funding acquisition, supervision, and writing–review and editing. **Rini Yanti:** data interpretation and validation. **Ulyatu Fitrotin:** data curation, formal analysis, funding acquisition, investigation, methodology, project administration, resources, software, validation, visualization, writing–original draft preparation, and writing–review and editing.

## Funding

This research was funded by the Productive‐Innovative Research Program, supervised by the Indonesia Endowment Fund for Education Ministry of Finance—Republic of Indonesia (grant number: PRJ‐72/LPDP/2019).

## Supporting Information

Figure S1: Analysis method for microbial contamination.

## Supporting information


**Supporting Information** Additional supporting information can be found online in the Supporting Information section.

## Data Availability

The data presented in this study are available upon request.
