# Supplementary figures and images for: Incorporation of Lactiplantibacillus plantarum subsp. plantarum Dad‐13 Into Chocolate Processing: The Effect on Physical, Nutritional, and Probiotics Viability During Storage
Source: Scientifica (Cairo). 2025 Dec 30;2025:5511985. doi: 10.1155/sci5/5511985 (PMC12782336; doi:10.1155/sci5/5511985)

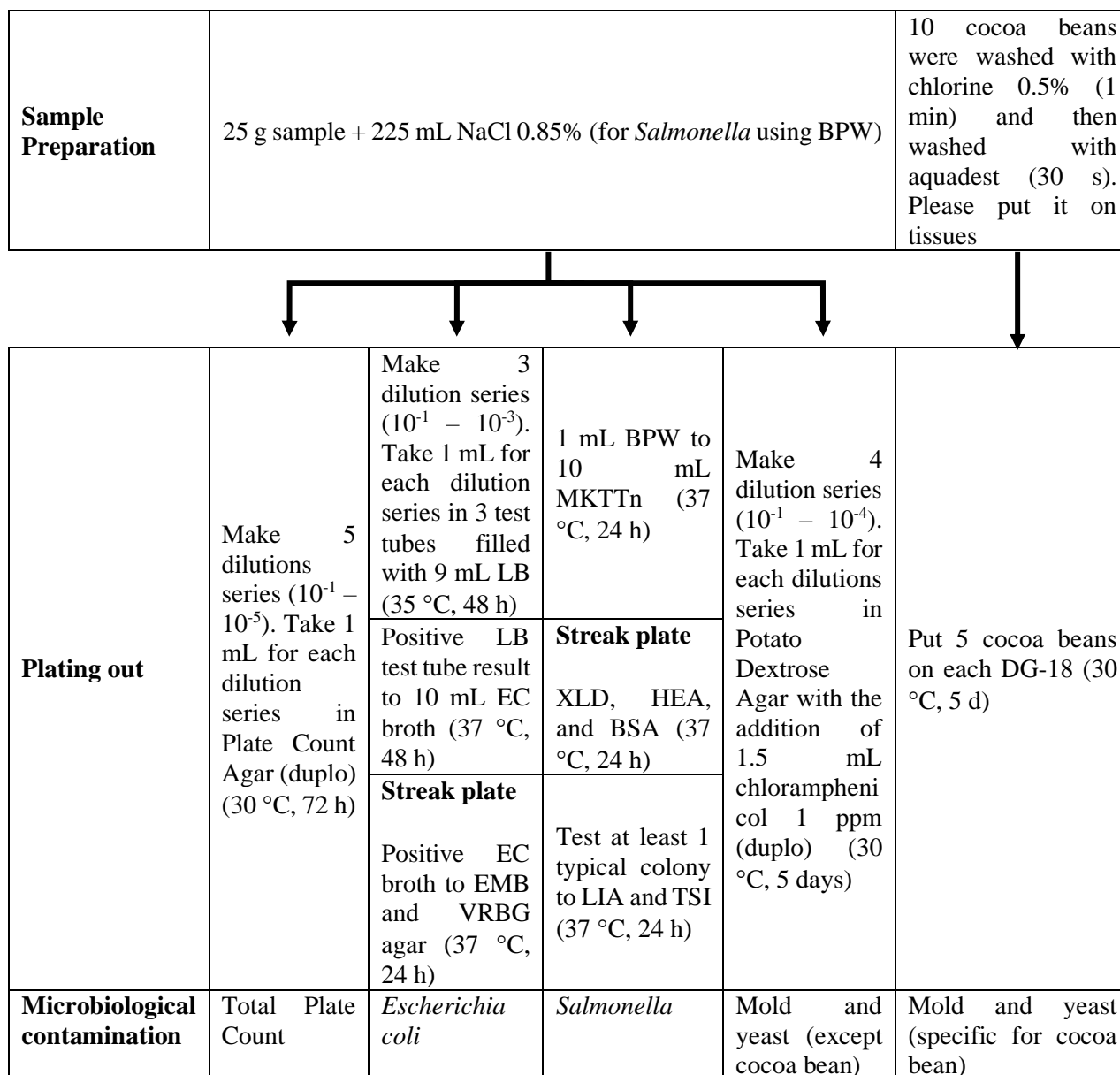

**Supplementary Figure S1.** Analysis method for microbiological contamination.

Supplement: Supplementary file 1 — Supporting Information Additional supporting information can be found online in the Supporting Information section. [file SCI5-2025-5511985-s001.pdf]
